# Clinical Correlations of Motor and Somatosensory Evoked Potentials in Neuromyelitis Optica

**DOI:** 10.1371/journal.pone.0113631

**Published:** 2014-11-25

**Authors:** Wei-Chia Tsao, Rong-Kuo Lyu, Long-Sun Ro, Ming-Fen Lao, Chiung-Mei Chen, Yih-Ru Wu, Chin-Chang Huang, Hong-Shiu Chang, Hung-Chao Kuo, Chun-Che Chu, Kuo-Hsuan Chang

**Affiliations:** 1 Department of Neurology, Chang Gung Memorial Hospital-Linkou Medical Center, Chang Gung University College of Medicine, Taoyuan, Taiwan; 2 Department of Neurology, Kaohsiung Medical University Hospital, Kaohsiung, Taiwan; University of Düsseldorf, Germany

## Abstract

**Background:**

Motor and somatosensory evoked potentials (MEPs and SSEPs) are sensitive tools for detecting subclinical lesions, assessing disease severity, and determining the prognosis for outcomes of patients with inflammatory neurological diseases such as multiple sclerosis. However, their roles in neuromyelitis optica (NMO), a severe inflammatory neurological disease that predominantly involves optic nerves and spinal cord, have not yet been clarified.

**Methods and Findings:**

Clinical symptoms and examination findings at relapses of 30 NMO patients were retrospectively reviewed. Abnormal MEPs were observed in 69.2% of patients. Patients with abnormal motor central conduction time (CCT) of the lower limbs had higher Kurtzke Expanded Disability Status Scale (EDSS) scores than those with normal responses (*P* = 0.027). Abnormal SSEPs were found in 69.0% of patients. Patients with abnormal lower limb sensory CCT had higher EDSS scores than those with normal responses (*P* = 0.019). In 28 patients followed up more than 6 months, only one of 11 patients (9.1%) with normal SSEPs of the lower limbs had new relapses within 6 months, whereas 8 of 17 patients (47.1%, *P* = 0.049) with abnormal SSEPs of the lower limbs had new relapses.

**Conclusions:**

These results indicate MEPs and SSEPs of the lower limbs are good indicators for the disability status at relapses of NMO. Lower limb SSEPs may be a good tool for reflecting the frequency of relapses of NMO.

## Introduction

Motor and somatosensory evoked potentials (MEPs and SSEPs) can play a role in the assessment of many inflammatory neurological diseases. For example, recording lower limb SSEPs is a sensitive technique for detection of clinical abnormalities in patients with multiple sclerosis (MS) [1]. Lines of evidence demonstrate a high yield of MEP abnormal findings in MS patients [2], [3]. MEPs and SSEPs have been reported to be predictive of later clinical disability either when taking into account the timing of electrophysiological examinations in relation to MS patients' individual disease course [4], [5], or after using multimodal visual, auditory, somatosensory, and motor evoked potentials to evaluate MS patients [5]. MEP and SSEP abnormalities also provide evidence of long tract damage in acute disseminated encephalomyelitis and post-infectious myelitis [6]–[8]. Therefore, MEPs and SSEPs are thought as easy and sensitive tools to detect lesions that may not always been revealed by neuroimaging studies.

Neuromyelitis optica (NMO) is an inflammatory disease mainly characterized by optic neuritis (ON) and longitudinal extended spinal cord lesions (LESCLs) [9]. It frequently displays a relapsing-remitting course similar to that of MS, and was frequently classified as optico-spinal MS (OSMS) before the development of its own biomarker, anti-aquaporin-4 (AQP4) antibody [10]. Anti-AQP4 antibody is seen in around 61%–90% of patients with NMO, while only 0%–9% of MS patients have this antibody [10]–[13]. In NMO, spinal cord involvement often presents in the form of complete transverse myelitis with para- or tetraparesis, an almost symmetrical sensory level, and sphincter dysfunction [14], [15]. In contrast, spinal cord symptoms in MS are milder and asymmetric, and are caused by acute partial transverse myelitis [16]. These clear distinctions suggest that NMO and MS could be two different CNS inflammatory diseases.

EPs are convenient tools that could sensitively detect subclinical abnormalities that are not captured by neuroimaging studies [17]. Therefore EPs could provide valuable information about disease activity and are widely applied in daily clinical practice. There have been a limited number of studies that have reported on MEPs and SSEPs in NMO [18]. It has been shown that SSEPs are abnormal in 85.7% of Cuban patients with NMO [19]. A Japanese study found that 4 of 9 OSMS patients have prolonged SSEPs [20]. To further understand the clinical role of MEPs and SSEPs in NMO, we correlated the results of MEP and SSEP with other clinical information including spinal cord magnetic resonance imaging (MRI), and the degree of disability in the acute and remission stages of NMO relapses. Our findings indicate the important value of MEPs and SSEPs in evaluating clinical disability and predicting relapse recurrence of NMO patients.

## Materials and Methods

### Ethics statement and study populations

We retrospectively reviewed the records of all hospitalized patients with NMO in Chang Gung Memorial Hospital-Linkou Medical Center from January 2011 to September 2013, and found 40 patients diagnosed with NMO according to Wingerchuk's criteria published in 2006 [9]. Anti-AQP4 antibody assay was performed in all of these patients. Ethics approval was provided by the institutional review boards of the Chang Gung Memorial Hospital (ethical license No: 100-1083B). Written informed consent was obtained from each patient before they were examined.

A relapse of NMO was defined as the occurrence, recurrence, or worsening of symptoms of neurological dysfunction that lasted >24 hours and then stabilized or eventually resolved, either partially or completely [21]. Symptoms that occurred within one month after the initial symptoms at relapse were considered to be part of the same episode [21], MEPs and/or SSEPs that were recorded within 30 days after the initial symptoms of relapse were selected for further analysis. Duration of disease from onset to EPs was determined either by reported clinical signs suggestive of NMO or by establishing the diagnosis through appropriate history and examination. If two or more EPs were collected from one patient, we analyzed the earliest EP results after corresponding relapses. Kurtzke Expanded Disability Status Scale (EDSS) scores and Kurtzke's functional system (FS) scores were evaluated at relapses and 6 months later [22]. A corticosteroid (1000 mg methylprednisone administered intravenously for five consecutive days) was prescribed in the acute stage of relapses, and all patients received low dose prednisolone (≤0.5 mg/kg/day) in the remission stage. Azathioprine or mycophenolate were also prescribed in some patients in combination with a low dose steroid.

### Evoked potentials

Motor evoked potential and SSEP responses were obtained from the affected side. Normal values were collected from 50 healthy individuals (26 males and 24 females; mean age, 56.3±11.8 years) from previous published data from our hospital [23]. The results were considered abnormal if either latencies or central conduction time (CCT) exceeded normal limits, or were absent.

#### Motor evoked potentials

Motor evoked potentials were evoked by using a Magstim nerve stimulator (Magstim, Dyfed, UK) and the responses were acquired and analyzed with a Nicolet Viking System (Nicolet Biomedical, Madison, WI, USA). MEPs were obtained from the abductor pollicis brevis (APB) muscles for the upper limbs and the tibialis anterior (TA) muscles for the lower limbs. For the upper limbs, magnetic stimuli were applied to the Cz position of the 10–20 international system of EEG electrode placement for cortical stimulation and the C7 vertebra for peripheral motor stimulation. For the lower limbs, motor cortex (2–4 cm more frontal than upper limbs stimulation) and the fourth lumbar root (L4) were elicited. We assessed MEP latencies, and the motor CCT was calculated as the difference between cortical motor latency and peripheral motor latency.

#### Somatosensory evoked potentials

Somatosensory evoked potential studies were completed by using a Nicolet Viking System (Nicolet Biomedical, Madison, WI, USA). For recording the median SSEP, surface electrodes were placed on Erb's point, the second spine, and the scalp overlying the primary sensory area in the parietal lobe contralateral to the stimulated limb (2 cm behind the 10–20 system, C3 and C4 locations). A reference electrode was placed on Fz. The median nerve was stimulated at each wrist using 0.2 ms square wave electrical pulses. The stimulus intensity was adjusted to produce a visible twitch of the APB muscle without causing discomfort. For the tibial nerve, the SSEP was recorded on the L2 spinous process and on the midline of the scalp 2 cm posterior to the vertex. The tibial nerve was stimulated at the ankle. At least 500 responses were averaged for each test. To confirm SSEP reproducibility, each measurement was repeated at least twice. The following measurements were recorded for the median SSEP: peak latency of responses recorded at Erb's point (N9), the C2 spinous process (N13), and the scalp (N20). Upper limb sensory CCT between N13 and N20 was calculated. In the tibial SSEP, the peak latency of responses at the L2 spinous process (N22) and the scalp (P40), as well as lower limb sensory CCT between N22 and P40, were recorded.

### Magnetic resonance imaging

Magnetic resonance imaging scans were obtained at the time of clinical relapse (2–21 days from relapse), with protocols including T1-, T1-enhanced, and T2-weighted images. The length of the spinal cord lesions was expressed in terms of the number of vertebral segments. Radiologists who were blinded to the study reported the results of all MRIs.

### Statistical analysis

The Statistical Program for Social Sciences (SPSS) statistical software (version 19.0, Chicago, IL, USA) was used for statistical analysis. *P* values<0.05 were considered statistically significant, and all *P* values were two-tailed. Comparisons of EP variance with respect to the demographic and clinical data were conducted by using Student's *t* test. Correlations between EDSS scores and spinal segment involvement were calculated as Spearman's rank correlation. Categorical variables were compared using Chi-Square analysis or Fisher's exact test, when either was appropriate. The association between EPs and a relapse after adjustment with designate variables was computed based on a logistic regression with random intercept, the significant level was set at 5%.

## Results

**Figure 1:**
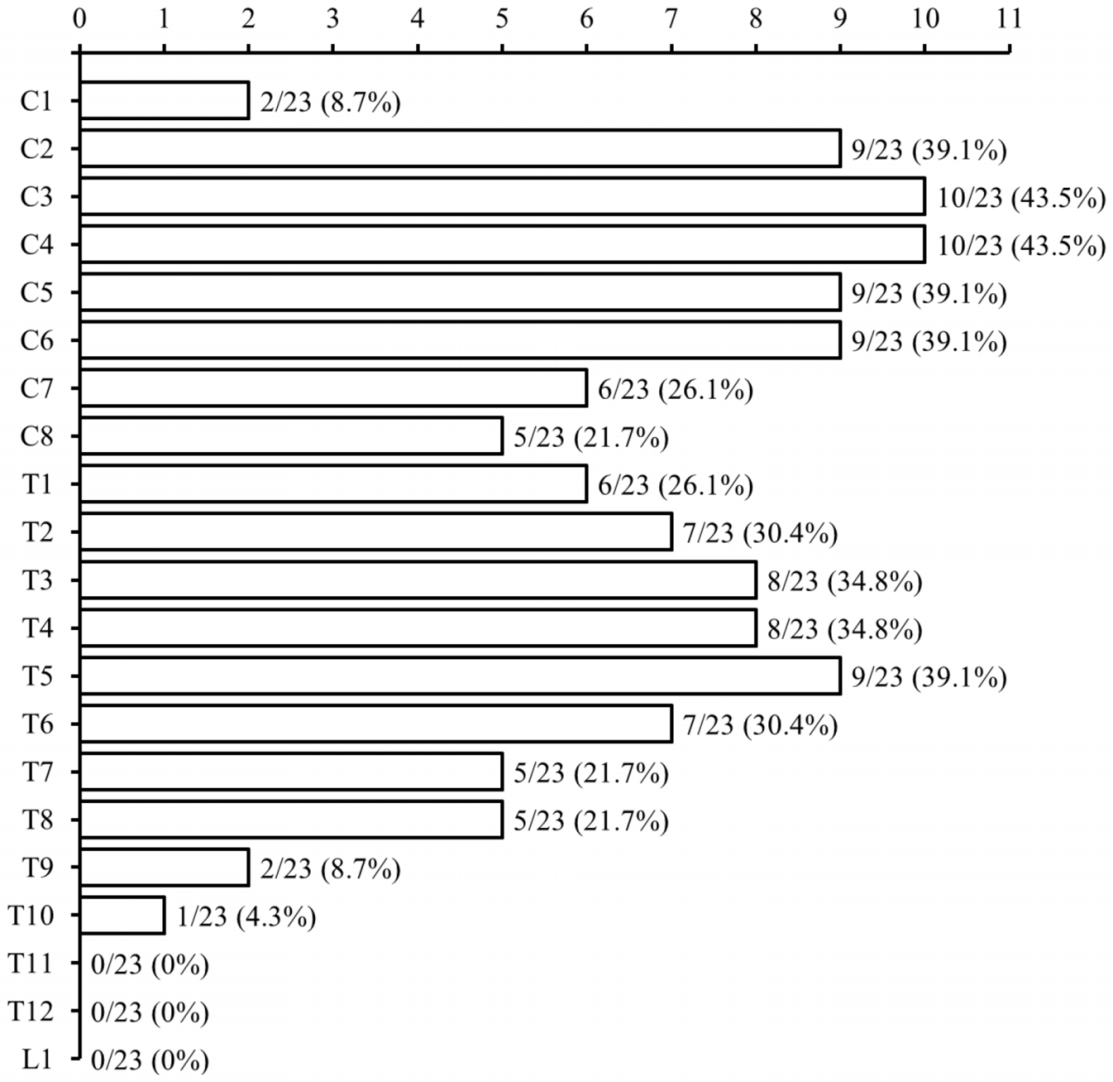
Distribution of spinal cord affected segments in neuromyelitis optica patients (n = 23).

Of the 40 NMO patients, 30 had MEP and/or SSEP studies and were recruited for further analysis. Twenty-five patients had both MEP and SSEP studies, 4 had only SSEP, and one patient had only MEP at the acute stage of relapse. The most frequent functional abnormality of NMO relapses was abnormal sensation (**Table 1**, 90.0%), followed by weakness (83.3%), bowel dysfunction (40.0%), and blurred vision (23.3%). The mean EDSS at relapse was 5.4±1.8, the mean Kurtzke's pyramidal FS score was 3.3±1.9, and the mean somatosensory FS score was 3.3±1.3. There was no significant correlation between segmental lengths and EDSS score. Almost an equal number of patients had only cervical or thoracic segment involvement (26.1% and 30.4%, respectively), while 43.5% of patients had both cervical and thoracic segments involved. The most frequently involved spinal segment was C3/4 (43.5%), followed by C2, C5/6 (39.1%) and T5 (39.1%) (**Figure 1**).

**Table 1:** Demographic and clinical features of neuromyelitis optica patients.

	Patients
Male∶Female	2∶28
Mean age (years ± SD)	46.7±13.3
Disease duration (years ± SD)	4.1±4.4
Clinical presentations at relapse (%)	
Abnormal sensation^a^	27/30 (90.0%)
Weakness	25/30 (83.3%)
Ataxia and brain stem signs^b^	3/30 (10.0%)
Sphincter dysfunction	12/30 (40.0%)
Blurred vision	7/30 (23.3%)
EDSS at relapse	5.4±1.8
Presence of AQP4 antibody (%)	28/30 (93.3%)
Spinal cord lesion distribution	
Only cervical spinal cord involved	6/23 (26.1%)
Only thoracic spinal cord involved	7/23 (30.4%)
Both cervical and thoracic involved	10/23 (43.5%)
Lengths of involved spinal segments (± SD)	5.1±2.3

EDSS: Kurtzke Expanded Disability Status Scale; SD, standard deviation.

a Abnormal sensation including impairment of superficial sensation, and loss of sense of passive movement and vibration.

b Ataxia and brain stem signs including dizziness, ataxia, dysmetria, diplopia, dysarthria, dysphagia, hiccups, and nystagmus.

Abnormal MEPs were observed in 18 (69.2%) patients (**Table 2**). The frequencies of absent or prolonged motor CCT of the lower limbs (69.2%) were significantly higher than those in the upper limbs (38.5%, *P* = 0.026). All patients with abnormal MEPs of the upper limbs had abnormal MEPs of the lower limbs as well. Patients who had abnormal cortical latency (CxL) of the lower limbs had higher EDSS scores (6.2±1.7) than those with normal responses (4.5±1.6, *P* = 0.014). Similarly, patients with abnormal motor CCT of lower limbs had higher EDSS scores (5.9±1.8) than those with normal responses (4.3±1.5, *P* = 0.027). The Kurtzke's pyramidal FS scores were also higher in patients with abnormal CxL of lower limbs (*P* = 0.016) and lower limb motor CCT (*P* = 0.029) than those with normal responses. Abnormal SSEPs were found in 20 (69.0%) patients (**Table 3**). The EDSS scores of the patients with abnormal P40 responses (6.0±1.6) were significantly higher than those of patients with normal responses (4.5±1.8, *P* = 0.026). Patients with abnormal sensory CCT of the lower limbs had higher EDSS scores (6.0±1.6) than those with normal responses (4.5±1.8, *P* = 0.019). The Kurtzke's somatosensory FS scores were also higher in patients having abnormal P40 (*P* = 0.036) and sensory CCT of lower limbs (*P* = 0.006) than those having normal responses. Both patients with abnormal and normal MEP/SSEPs of the lower limbs displayed identical age of onset, duration from onset to EPs, and length of involved segments on spinal MRI. There was no significant difference between abnormal and normal MEP/SSEPs of the upper limbs with respect to EDSS and Kurtzke's FS scores and the above parameters as well.

**Table 2:** Comparison of clinical features between neuromyelitis optica patients with normal and abnormal motor evoked potentials.

Motor evoked potentials (n = 26)	Number of patients (%)		Age at onset (years ± SD)	Duration from onset to time of study (years ± SD)	EDSS (± SD)	Length of involved spinal segments (± SD)
Upper limb CxL						
Normal	20	(76.9)	49.6±12.7	5.1±4.5	5.4±1.7	5.2±1.9
Prolonged/Absent	6	(23.1)	47.8±12.2	2.0±2.1	5.6±2.3	3.3±1.9
Upper limb motor CCT						
Normal	16	(61.5)	49.9±12.6	5.9±4.5	5.2±1.7	4.8±2.0
Prolonged/Absent	10	(38.5)	48.0±12.5	1.9±2.0	5.9±2.0	4.8±2.2
Lower limb CxL						
Normal	12	(46.2)	47.0±10.0	4.4±4.4	4.5±1.6	3.7±2.0
Prolonged/Absent	14	(53.8)	51.0±14.2	4.4±4.2	6.2±1.7^a^	5.4±1.9
Lower limb motor CCT						
Normal	8	(30.8)	50.6±9.3	5.8±4.8	4.3±1.5	4.3±2.2
Prolonged/Absent	18	(69.2)	48.5±13.7	3.8±3.9	5.9±1.8^b^	4.9±2.1

CCT: central conduction time; CxL: cortical latency; EDSS: Kurtzke Expanded Disability Status Scale; SD, standard deviation.

a *P* = 0.014, compared with normal responses;

b *P* = 0.027, compared with normal responses.

**Table 3:** Comparison of clinical features between neuromyelitis optica patients with normal and abnormal somatosensory evoked potentials.

Somatosensory evoked potentials (n = 29)	Number of patients (%)		Age at onset (years ± SD)	Duration from onset to time of study (years ± SD)	EDSS (± SD)	Length of involved spinal segments (± SD)
N20						
Normal	18	(62.1)	45.8±14.7	4.2±4.1	5.0±1.8	5.4±2.3
Prolonged/Absent	11	(37.9)	49.6±10.7	4.4±5.0	6.0±1.7	5.1±2.3
Upper limb sensory CCT						
Normal	18	(62.1)	47.3±13.9	4.3±4.0	5.1±1.9	5.6±2.3
Prolonged/Absent	11	(37.9)	47.2±12.8	4.1±5.2	5.8±1.8	4.9±2.3
P40						
Normal	12	(41.4)	43.0±12.4	4.0±4.8	4.5±1.8	4.9±2.0
Prolonged/Absent	17	(58.6)	50.2±13.3	4.4±4.2	6.0±1.6^a^	5.5±2.4
Lower limb sensory CCT						
Normal	12	(41.4)	43.7±12.4	3.8±4.8	4.5±1.8	4.9±2.0
Prolonged/Absent	17	(58.6)	49.8±13.5	4.5±4.2	6.0±1.6^b^	5.5±2.4

CCT: central conduction time; EDSS: Kurtzke Expanded Disability Status Scale; SD, standard deviation.

a *P* = 0.026, compared with normal responses;

b *P* = 0.019, compared with normal responses.

**Figure 2:**
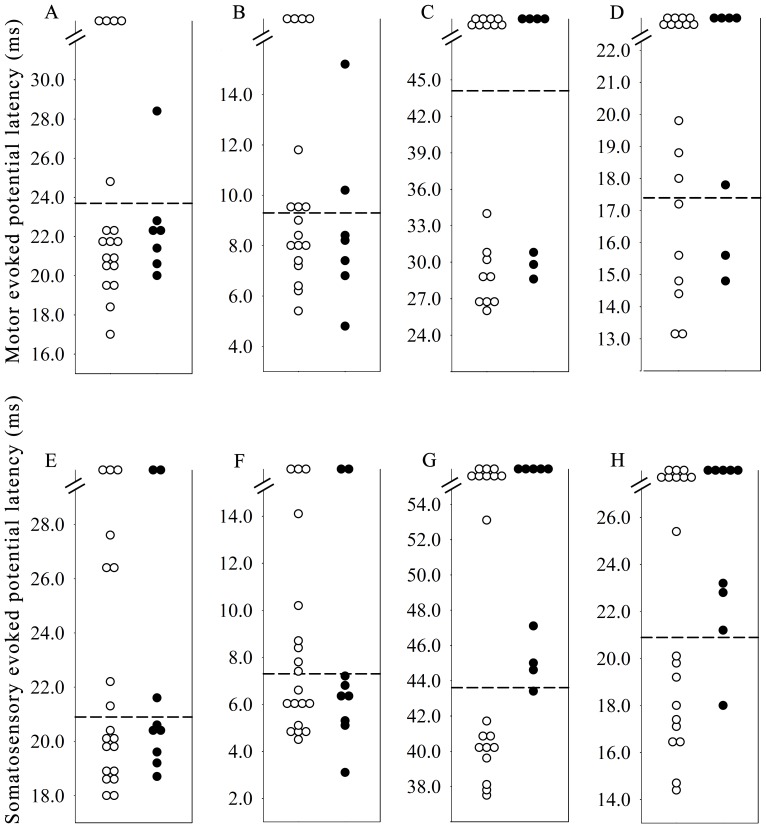
Scatter plots showing latencies of motor evoked potentials and somatosensory evoked potentials of NMO patients. A. CxL in upper limbs; B, upper limb motor CCT; C, CxL in lower limbs; D, lower limb motor CCT; E, N20; F, upper limb sensory CCT; G, P40; H, lower limb sensory CCT. Black dots indicate patients with a relapse. Note in G (P40) and H (lower limb sensory CCT), among 17 patients with abnormal evoked potentials (dots above the dashed lines) 8 patients (47.1%) had a relapse within 6 months after the index event.

To further explore the clinical correlation between MEPs/SSEPs and follow-up outcome in NMO, we calculated the changes in EDSS scores between acute (at the onset) and chronic remission stages (after 6 months follow-up) of documented episodes. One patient was lost to follow-up and excluded from this analysis. Values of MEP and SSEP onset latencies were plotted for in **Figure 2**. We did not see any significant difference in changes of EDSS scores between patients with normal and abnormal MEPs/SSEPs (**Table 4** and **Table 5**). However, new relapses were noted in nearly half of patients with abnormal sensory CCT and P40 responses of the lower limbs (47.1%), whereas only one patient (9.1%, *P* = 0.049) with normal P40 responses and sensory CCT of the lower limbs had relapse within 6 months after the onset of the index episode (Odds ratio = 8.3). Prolonged lower limb sensory CCT and P40 increased the odds of a relapse within 6 months after index event by a factor of 3.4 (*P* = 0.029) after adjusting ages of onset, gender and uses of immunosuppressants.

**Table 4:** Comparison of changes of EDSS scores and frequencies of relapses within 6 months between neuromyelitis optica patients with normal and abnormal motor evoked potentials.

	Number of patients	EDSS (± SD)			New relapses within 6 months	Immunosuppressants^a^
		At relapse	6 months after relapse	Change		
Upper limb CxL						
Normal	19	5.3±1.7	3.8±2.2	−1.5±1.5	6/19 (31.6%)	2/19 (10.5%)
Prolonged/absent	6	5.6±2.3	4.3±3.1	−1.3±1.3	1/6 (16.7%)	2/6 (33.3%)
Upper limb motor CCT						
Normal	15	5.0±1.7	3.8±2.2	−1.3±1.2	5/15 (33.3%)	2/15 (13.3%)
Prolonged/absent	10	5.9±2.0	4.1±2.7	−1.8±1.7	2/10 (20.0%)	2/10 (20.0%)
Lower limb CxL						
Normal	12	4.5±1.6	2.9±1.8	−1.6±1.2	3/12 (25.0%)	3/12 (25.0%)
Prolonged/absent	13	6.2±1.8^b^	4.8±2.5^c^	−1.3±1.7	4/13 (30.8%)	1/13 (7.7%)
Lower limb motor CCT						
Normal	8	4.3±1.5	2.8±1.6	−1.4±1.2	2/8 (25.0%)	2/8 (25.0%)
Prolonged/absent	17	5.9±1.8^d^	4.4±2.5	−1.5±1.6	5/17 (29.4%)	2/17 (11.8%)

CCT: central conduction time; CxL: cortical latency; EDSS: Kurtzke Expanded Disability Status Scale; SD, standard deviation.

a Azathioprine or mycophenolate;

b *P* = 0.022, compared with normal responses;

c *P* = 0.037, compared with normal responses;

d *P* = 0.036, compared with normal responses.

**Table 5:** Comparison of changes of EDSS scores and frequencies of relapses within 6 months between Kurtzke Expanded Disability Status Scale patients with normal and abnormal somatosensory evoked potentials.

	Number of patients	EDSS (± SD)			New relapses within 6 months	Immunosuppressants^a^
		At relapse	6 months after relapse	Change		
N20						
Normal	17	4.9±1.8	3.6±2.4	−1.4±1.1	6/17 (35.3%)	2/17 (11.8%)
Prolonged/absent	11	6.0±1.7	4.2±2.3	−1.7±1.7	3/11 (27.3%)	1/11 (9.1%)
Upper limb sensory CCT						
Normal	17	5.0±1.8	3.5±2.4	−1.5±1.1	7/17 (41.2%)	2/17 (11.8%)
Prolonged/absent	11	5.8±1.8	4.4±2.2	−1.5±1.7	2/11 (18.2%)	1/11 (9.1%)
P40						
Normal	11	4.3±1.7	2.3±1.2	−2.0±0.8	1/11 (9.1%)	2/11 (18.2%)
Prolonged/absent	17	6.0±1.6^b^	4.8±2.3^c^	−1.2±1.5	8/17 (47.1%)^d^	1/17 (5.9%)
Lower limb sensory CCT						
Normal	11	4.2±1.7	2.2±1.3	−2.0±0.9	1/11 (9.1%)	2/11 (18.2%)
Prolonged/absent	17	6.0±1.6^e^	4.9±2.2^f^	−1.2±1.5	8/17 (47.1%)^g^	1/17 (5.9%)

CCT: central conduction time; EDSS: Kurtzke Expanded Disability Status Scale; SD, standard deviation.

a Azathioprine or mycophenolate;

b *P* = 0.012, compared with normal responses;

c *P* = 0.001, compared with normal responses;

d *P* = 0.049, compared with normal responses;

e *P* = 0.008, compared with normal responses;

f *P* = 0.001, compared with normal responses;

g *P* = 0.049, compared with normal responses.

## Discussion

Our results showed that 69.2% and 69.0% of NMO patients hospitalized in our tertiary university center for severe relapses had abnormal MEPs and SSEPs at relapse, respectively. Patients with abnormal MEPs of the lower limbs demonstrated higher EDSS and Kurtzke's pyramidal FS scores than those with normal responses. Higher EDSS and Kurtzke's somatosensory and pyramidal FS scores were also noted in the patients with abnormal SSEPs of the lower limbs compared with patients with normal responses. Patients with normal SSEPs of the lower limbs had lower probability of developing new relapses during the following 6 months than those with abnormal responses. These results indicate that lower limb MEPs and SSEPs are good indicators of the disability status and relapsing activity in NMO patients.

Motor evoked potentials and SSEPs are good indicators for monitoring white matter lesions of the spinal cord. In animal and human studies, selective ablation of the dorsal columns attenuates or abolishes the SSEP, which reveals that within the spinal cord SSEPs are mediated predominantly via the dorsal columns [24]. Lines of evidence in lesioning studies show that most of the MEP signals travel in the area of the corticospinal tract at the lateral columns [25]. Although conventional MRI suggests the predominant involvement of central grey matter in the spinal cord [26], [27], MRI studies by diffusion tensor imaging have found significant white matter damage of spinal cord in patients with NMO [28], [29]. Pathological studies also demonstrate that the inflammation, necrosis, and cavitation in NMO affect both the grey and white matter in spinal cord [14], [30], [31]. The high proportion of abnormal CCTs in MEPs and SSEPs further indicate that these white matter lesions in NMO interfere with the conduction of the long tracts of both the motor and sensory systems in the CNS. Given that involvement of thoracic segments is noted in 73.9% of NMO patients, it is not surprising that lower limb MEPs and SSEPs have a higher probability of being affected than upper limb MEPs and SSEPs. These results are similar to those in MS patients, showing that lower limb MEPs and SSEPs are more sensitive than upper limb MEPs and SSEPs [1], [3], [32].

Our results showing higher EDSS scores at relapses of NMO with abnormal MEPs or SSEPs indicate the potential of these electrophysiological tests in evaluating clinical disability. Although this finding has not been reported in NMO before, the correlations between EPs and clinical disability have been shown in other neurological diseases such as MS [33]–[35], spondylotic myelopathy [23], [36], vitamin B12 deficiency [37], and hereditary spastic paraplegia [38]. Performing EP studies in MS patients is thought to have the potential to reveal subclinical organic lesions or indicate lesions from vague complaints, and thus may help establish evidence of relapses [33], or have the potential to be used as a tool for disease burden follow-up [34] and identification of patients with poor prognosis [35]. In spondylotic myelopathy, normal preoperative SSEPs in the upper limbs can be an important indicator for better postoperative outcomes [23], [36]. In patients with vitamin B12 deficiency, upper limb SSEPs correlate with disease duration, and a clear correlation is found between lower limb SSEPs and serum vitamin B12 level [37].

The factors to determine clinical outcomes of NMO were reported in previous literatures. A relapsing course demonstrates a poorer prognosis than monophasic course [15]. More brain lesions on MRI are indicative of poor visual acuity outcomes [39]. We found that SSEPs of the lower limbs may be useful for identifying NMO patients with high recurrent probabilities. Slow tapering of steroid and early immunosuppressive therapy may be needed in patients with abnormal SSEPs of the lower limbs. It would be also important to know whether these two groups of patients should be treated with different dosages of disease modifying therapies, such as rituximab [40]–[42] and tocilizumab [43], [44]. On the other hand, MEPs and SSEPs at relapses are not correlated with EDSS recovery in NMO patients, which could be affected by treatment of steroid or immunosuppressant. In MS, EPs at relapses are less correlated with disease disability than those during remission [45]. Thus analysis of EPs during remission may demonstrate correlation with disability recovery in NMO.

Our study has several limitations. It has inherent bias because of its retrospective study design, and the number of patients was relatively small. EDSS score may not appropriately indicate the degree of disability, especially in the patients without a walking problem. All of the EP studies were performed in severe relapses that cause hospitalization, and therefore EPs in some minor relapses, such as minor optic neuritis, may be missed. The low rate of patients under immunosuppressant therapy may be helpful to reveal the natural electrophysiological feature of NMO, whereas may also influences the transferability of the results to the patients with appropriate disease modifying therapy. Nevertheless, our findings support the role of MEP and SSEP studies for evaluating clinical disability and recurrent relapses in NMO patients. Further prospective studies with a larger number of patients along with longer cohort studies to identify the electrophysiological characteristics of NMO patients are warranted.
